# Origin and Evolution of Sulfadoxine Resistant *Plasmodium falciparum*

**DOI:** 10.1371/journal.ppat.1000830

**Published:** 2010-03-26

**Authors:** Sumiti Vinayak, Md Tauqeer Alam, Tonya Mixson-Hayden, Andrea M. McCollum, Rithy Sem, Naman K. Shah, Pharath Lim, Sinuon Muth, William O. Rogers, Thierry Fandeur, John W. Barnwell, Ananias A. Escalante, Chansuda Wongsrichanalai, Frederick Ariey, Steven R. Meshnick, Venkatachalam Udhayakumar

**Affiliations:** 1 Atlanta Research and Education Foundation, Atlanta, Georgia, United States of America; 2 Malaria Branch, Division of Parasitic Diseases, National Center for Zoonotic Vector Borne and Enteric Diseases, Coordinating Center for Infectious Diseases, Centers for Disease Control and Prevention, Atlanta, Georgia, United States of America; 3 National Malaria Center, Phnom Penh, Cambodia; 4 US Naval Medical Research Unit No. 2, Jakarta, Indonesia; 5 Department of Epidemiology, UNC School of Public Health, Chapel Hill, North Carolina, United States of America; 6 Institut Pasteur in Cambodia, Phnom Penh, Cambodia; 7 Institut Pasteur, Unité d'Immunologie Moléculaire des Parasites, Paris, France; 8 School of Life Sciences, Arizona State University, Tempe, Arizona, United States of America; Washington University School of Medicine, United States of America

## Abstract

The Thailand-Cambodia border is the epicenter for drug-resistant falciparum malaria. Previous studies have shown that chloroquine (CQ) and pyrimethamine resistance originated in this region and eventually spread to other Asian countries and Africa. However, there is a dearth in understanding the origin and evolution of *dhps* alleles associated with sulfadoxine resistance. The present study was designed to reveal the origin(s) of sulfadoxine resistance in Cambodia and its evolutionary relationship to African and South American *dhps* alleles. We sequenced 234 Cambodian *Plasmodium falciparum* isolates for the *dhps* codons S436A/F, A437G, K540E, A581G and A613S/T implicated in sulfadoxine resistance. We also genotyped 10 microsatellite loci around *dhps* to determine the genetic backgrounds of various alleles and compared them with the backgrounds of alleles prevalent in Africa and South America. In addition to previously known highly-resistant triple mutant *dhps* alleles SGEGA and AGEAA (codons 436, 437, 540, 581, 613 are sequentially indicated), a large proportion of the isolates (19.3%) contained a 540N mutation in association with 437G/581G yielding a previously unreported triple mutant allele, SGNGA. Microsatellite data strongly suggest the strength of selection was greater on triple mutant *dhps* alleles followed by the double and single mutants. We provide evidence for at least three independent origins for the double mutants, one each for the SGKGA, AGKAA and SGEAA alleles. Our data suggest that the triple mutant allele SGEGA and the novel allele SGNGA have common origin on the SGKGA background, whereas the AGEAA triple mutant was derived from AGKAA on multiple, albeit limited, genetic backgrounds. The SGEAA did not share haplotypes with any of the triple mutants. Comparative analysis of the microsatellite haplotypes flanking *dhps* alleles from Cambodia, Kenya, Cameroon and Venezuela revealed an independent origin of sulfadoxine resistant alleles in each of these regions.

## Author Summary

Widespread resistance to chloroquine (CQ) and sulfadoxine-pyrimethamine (SP), the two least expensive and widely available antimalarial drugs, has become a major global public health challenge. It is known that point mutations in *Plasmodium falciparum crt*, *dhfr* and *dhps* genes contribute to resistance to CQ, pyrimethamine and sulfadoxine, respectively. CQ and pyrimethamine resistance spread to Africa and Asia from a few founding mutant lineages originating from the Thailand-Cambodia border. Here, we define the origins of *dhps* alleles in Cambodia and their relationships to African and South American counterparts. Three different triple mutant alleles including a novel allele comprised of 437G, 540N, and 581G mutations (S_436_ **G_437_N_540_G_581_**A_613_) were found in Cambodia as opposed to a single triple mutant allele in South America and two common double mutant alleles in Africa. Microsatellite data suggest strong selection operating on triple mutant alleles as compared to double and single mutants in Cambodia. We report three major independent origins for the double mutants and at least two independent origins for the highly resistant triple mutant *dhps* alleles in Cambodia. We also show that the resistant *dhps* alleles in Africa and South America have distinct origins from Cambodia. These results suggest that the evolution and spread of sulfadoxine resistance is different from CQ and pyrimethamine resistance.

## Introduction

The Thailand-Cambodia border in Southeast Asia has been an epicenter for drug resistant falciparum malaria where chloroquine (CQ) resistance emerged in the early 1960s [1], followed by sulfadoxine-pyrimethamine (SP) resistance in the late 1970s [2],[3] and mefloquine (MQ) resistance in the mid 1990s [4]. Following the emergence of MQ resistance, artemisinin-based combination therapy (ACT) consisting of artesunate (AS) and MQ was adopted as the first-line treatment against uncomplicated falciparum malaria in Thailand (1995) and Cambodia (2000) [4],[5].

Sulfadoxine-pyrimethamine (SP) combination is the most widely used antifolate to treat CQ-resistant falciparum malaria and, because of their synergistic effect, the combination is more effective than either drug used alone [6],[7],[8]. It is also one of the partner drugs of artemisinin-based combination therapy (ACT) currently being used for treatment of uncomplicated falciparum malaria in some parts of the world [9]. Importantly, SP is the only drug recommended by the World Health Organization (WHO) for intermittent preventive treatment (IPT) in pregnant women in Sub-Saharan Africa where large number of deaths occur due to malaria in pregnancy [9]. Sulfadoxine acts by inhibiting dihydropteroate synthase (*dhps*), an essential enzyme of the folate biosynthesis pathway [10]. Previous studies have identified mutations at five DHPS codons (S436A/F; A437G; K540E; A581G; and A613S/T) to be associated with sulfadoxine resistance in *P. falciparum* [11],[12],[13],[14],[15]. Kinetic studies have shown that the mutant DHPS enzyme has a reduced affinity for sulfadoxine, resulting in the development of resistance, the level of which broadly correlates with the number of mutations in *dhps* [13].

In Southeast Asia, at least two different triple mutant *dhps* alleles (AGEAA and SGEGA) have been described [16],[17],[18] whereas in South America, a single triple mutant allele SGEGA has been reported [19],[20],[21],[22],[23]. Interestingly in Africa, the SGEAA (east Africa) and AGKAA (west and central Africa) double mutants are the common *dhps* alleles [24],[25], with no triple mutant *dhps* alleles reported on this continent thus far. Similarly, the pyrimethamine-resistant quadruple mutant *dhfr* allele (51I/59R/108N/164L) is abundant in Southeast Asia and in low frequency in Africa [26],[27]. The triple mutant *dhfr* allele 51I/59R/108N is widespread in Southeast Asia and Africa, whereas in South America (mostly in the Peruvian Amazon) two other forms of the triple mutant (50R/51I/108N and 51I/108N/164L) are prevalent.

Microsatellite analysis has revealed that triple (51I/59R/108N) mutant *dhfr* alleles in Africa have shared ancestry with *dhfr* alleles from Southeast Asia suggesting that, like CQ resistance, the alleles conferring pyrimethamine resistance were also introduced into Africa from Southeast Asia [25],[26],[27],[28],[29],[30],[31],[32],[33]. However, unlike Southeast Asia where all *dhfr* alleles have single common ancestor [26], additional local evolution of the *dhfr* alleles has been reported in Africa [25],[27],[28],[34],[35]. In South America, the pyrimethamine-resistant *dhfr* alleles are thought to have two independent origins [20],[21],[22]. Recently, another independent origin of the double mutant *dhfr* allele (59R/108N) has been observed in Papua New Guinea [36]. Thus, similar to CQ resistance, at least four major distinct origins of pyrimethamine resistance have been reported worldwide.

Although the origins of CQ and pyrimethamine resistance are better understood, there is limited data related to the origins of sulfadoxine resistance globally. Previous studies have reported multiple and independent origins of double mutant *dhps* alleles in Africa [24],[25],[37], and one common origin for the triple mutant *dhps* allele in South America [20],[21],[22]. Importantly, the origins of the three different double mutants (AGKAA, SGKGA and SGEAA) and two different triple mutant (AGEAA and SGEGA) *dhps* alleles remain unknown in Southeast Asia. Therefore, the present study was aimed to (i) determine whether resistant *dhps* alleles in Cambodia are experiencing a selective sweep (ii) estimate the probable number of origins of the highly resistant *dhps* alleles in Cambodia and (iii) examine the evolutionary relationships between Southeast Asian (Cambodia), South American (Venezuela) and African (Cameroon and Kenya) *dhps* alleles.

## Materials and Methods

### Sample collection, DNA isolation and genotyping for *dhps* codons 436 to 613

**Figure 1:**
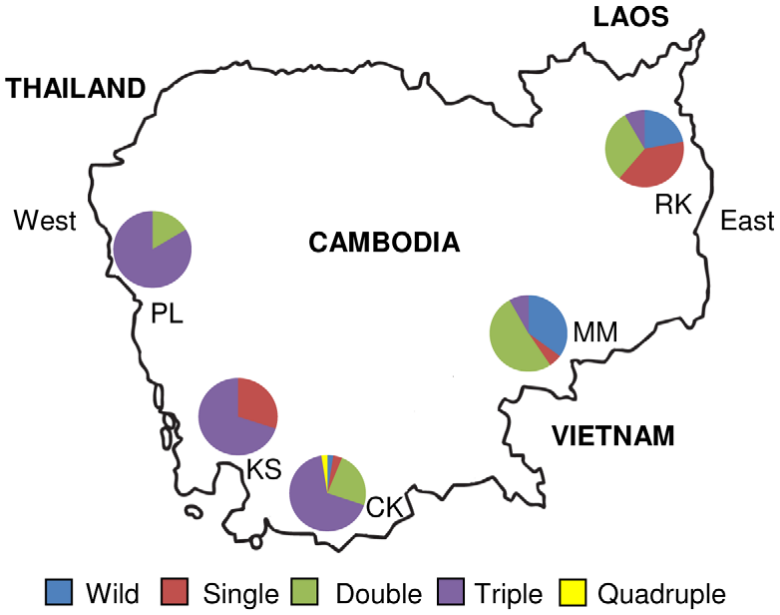
The map of Cambodia showing study sites. The pie-diagrams show the frequency distribution of the single, double, triple and quadruple mutant *dhps* alleles in Cambodia. PL, Pailin; KS, Kampong Seila; CK, Chumkiri; MM, Memut; RK, Rattanakiri.

*Plasmodium falciparum* clinical isolates were collected from patients with uncomplicated falciparum malaria from five sites in Cambodia: Pailin and Kampong Seila in the west, Chumkiri in the south, and Memut and Rattanakiri in the east (Fig. 1). Patients enrolled in this study were treated with ACT (Artesunate+Mefloquine) according to the national drug policy of Cambodia. Written informed consent was obtained from each patient before blood collection. The study was approved by Institutional Review Boards (IRBs) of the Cambodia National Ethics Committee for Health Research, the US Naval Medical Research Unit No. 2 (NAMRU-2) Jakarta, Indonesia, and the University of North Carolina at Chapel Hill (UNC), USA.

DNA was extracted from filter paper blood spots using QIAmp Mini kit (Qiagen, Valencia, CA, USA). A total of 234 *P. falciparum* isolates (Pailin, n = 51; Kampong Seila, n = 10; Chumkiri, n = 85; Memut, n = 43; and Rattanakiri, n = 45) were sequenced for a portion of the *dhps* gene covering codons 436, 437, 540, 581 and 613. Each DNA sample representing a clinical isolate was subjected to two rounds of PCR. The primary amplification was done using 5′-AACCTAAACGTGCTGTTCAA-3′ (Forward) and 5′-AATTGTGTGATTTGTCCACAA-3′ (Reverse) primers with the following cycling parameters: 5 minutes initial denaturation at 95°C followed by 35 cycles with 30 seconds denaturation at 95°C, 30 seconds annealing at 50°C, 1 minute extension at 68°C and a final 5 minute extension at 68°C. The primary amplification product was subjected to nested PCR using 5′-ATGATAAATGAAGGTGCTAG-3′ (Forward) and 5′-TCATTTTGTTGTTCATCATGT-3′ (Reverse) primers with the same cycling parameters as primary except that the annealing was done at 52°C and the number of cycles was reduced to 30. The 647 bp nested product was sequenced on an ABI 3130xl Genetic Analyzer (Applied Biosystems, Foster City, CA, USA).

### Microsatellite analysis

Parasite isolates with mixed *dhps* sequencing electropherograms (multiple peaks at one or more nucleotide) were removed from the microsatellite analysis. Only those with single *dhps* genotypes were assayed for 8 neutral microsatellite loci on chromosomes 2 (GenBank UniSTS ID: C2M27, C2M29, C2M34, C2M33) and 3 (GenBank UniSTS ID: C3M40, C3M88, C3M69 and C3M39) in order to exclude any additional multiply-infected samples and to obtain an estimate of the neutral baseline heterozygosity in the population [38]. These loci have previously been used for constructing a genetic map of *P. falciparum* and are not known to be under the influence of any selection [38]. PCR cycling parameters for all 8 neutral loci were adapted from Nair et al [26] as described earlier [21]. Isolates containing multiple alleles at one or more loci on chromosomes 2 and/or 3 were not carried forward for analyzing microsatellites on chromosome 8 around the *dhps* gene. Singly-infected isolates were typed for 10 microsatellite loci, five upstream (−11 Kb, −7.5 Kb, −2.9 Kb, −1.5 Kb, −0.13 Kb) and five downstream (0.03 Kb, 0.5 Kb, 1.4 Kb, 6.4 Kb and 9 Kb) of the *dhps* gene. Primer sequences and PCR cycling parameters for these loci are provided as supporting information available online (Table S1). The amplified PCR products were separated on ABI 3130xl Genetic Analyzer and analyzed using GeneMapper software v3.7 (Applied Biosystems, Foster City, CA, USA).

### Testing selection and genetic differentiation

To estimate the genetic variability at each of the 10 loci on chromosome 8 flanking *dhps*, we calculated number of alleles (A) as well as expected heterozygosity (*H_e_*) per locus using GenAlEx 6.2 [39]. *H_e_* was calculated using the formula (*H_e_*) = [*n*/(*n*−1)][1−Σ*p_i_^2^*], where *n* is the number of *P. falciparum* isolates genotyped for that locus and *p_i_* is the frequency of the *i*th allele. The sampling variance for *H_e_* was calculated as 2(n−1)/n^3^ {2(n−2) [Σ(*p_i_* ^3^−(Σ*p_i_* ^2^)^2^]}. In order to investigate whether a selective sweep had occurred around *dhps* alleles, we divided the entire sample set into groups based on either the number of mutations (wild type, single, double and triple mutant group) or type of mutations/genotype (e.g. SGEAA) in the *dhps* gene. Significant difference between the mean *H_e_* of any two groups was assessed using the Mann-Whitney *U* test implemented in the statistical package Stata version 8.1 for Windows (Stata Corporation, College Station, TX, USA). Differences were considered significant if the calculated *P* value was ≤0.05 (two-tailed test). Since microsatellite loci adjacent to a gene under selection (here *dhps*) may behave as non-neutral because of hitchhiking, we also performed similar analyses for 8 loci on chromosomes 2 and 3 which are putatively neutral.

We estimated F_ST_ between populations (following Weir and Cockerham) [40] at 10 *dhps* and 8 neutral microsatellite loci using the software GDA [41]. The populations were defined as wild, single, double and triple mutants according to the number of mutations in the *dhps* gene. 95% confidence intervals (CI) for each estimate and the standard deviation (SD) respectively, were computed by bootstrapping (over loci) and jackknifing (over population and loci) with 1000 permutations. To test whether F_ST_ at *dhps* loci differed from that of the neutral loci, we used Mann-Whitney *U* test.

It has been shown that loci directly under selection or linked to a selected loci exhibit extremely low or high levels of genetic differentiation between populations (F_ST_) as compared to neutral loci [42],[43],[44],[45],[46],[47],[48]. Such outlier loci can be identified by the coalescent simulations method of Beaumont and Nichols [43] where the F_ST_ distribution is plotted as a function of *H_e_* across loci under an island model of migration. We used this approach to disentangle selected from unselected loci, using FDIST program implemented in the software LOSITAN [49]. Simulations were initially run (under infinite-allele model) for only 8 neutral loci to calculate the mean neutral F_ST_ value which was subsequently used as “forced mean F_ST_” to run the second round of simulation along with all 10 *dhps* loci. The 95% confidence interval was achieved with 10,000 simulations of 4 expected populations with sample size of 50.

To assess whether there was underlying population structure that would bias our conclusions regarding selection, we used FSTAT 2.9.3.2 [50] to partition the variation in the population using both *dhps* and neutral loci. Here we considered each geographical site (Pailin, Chumkiri, Memut and Rattanakiri) as a discrete population to calculate F_ST_ (using 1000 permutations). Since there were only 7 singly-infected isolates from Kampong Seila and is located in western Cambodia, they were grouped along with Pailin.

The strength of linkage disequilibrium across all 10 loci around *dhps* alleles was estimated as standardized index of association (I_A_^S^), a haplotype-wide measure of linkage [51],[52] using LIAN version 3.5 [53]. I_A_^S^ was calculated using the formula I_A_^S^ = (V_D_/V_e_−1)/(L−1) where V_D_ is the observed mismatch variance, V_e_ is the expected mismatch variance and L is the number of loci tested. The null hypothesis of complete linkage equilibrium (I_A_^S^ = 0) was tested by the Monte-Carlo simulation process using 10,000 random permutations of the data.

### Determining the number of origins for *dhps* alleles

To examine the probable number of origins of sulfadoxine-resistant *dhps* alleles in Cambodia, a median-joining network was constructed using 10-loci haplotypes in NETWORK version 4.5.1.0 (http://www.fluxus-engineering.com/sharenet.htm). Median-joining networks are used for reconstructing the phylogeny of regions with reticulate evolution [54]. In order to understand the genetic relationships among the *dhps* alleles of Southeast Asia (Cambodia), Africa (Kenya and Cameroon) and South America (Venezuela) an eBURST (version 3) analysis [55] was performed. We used only 7 loci (−11 Kb, −7.5 Kb, −2.9 Kb, −0.13 Kb, 0.03 Kb, 0.5 Kb, 1.4 Kb) for this comparison as data for the other 3 loci were not available for African and South American isolates. Data for African and South American isolates were obtained from a previous study from our laboratory [37].

## Results

### Removal of multiply-infected samples

The region of the *dhps* gene containing codons 436, 437, 540, 581 and 613 was sequenced in 234 Cambodian *P. falciparum* isolates (Fig. 1). Out of these, 22 (∼9%) isolates contained mixed alleles at one or more *dhps* codons based on multiple peaks in the sequencing electropherograms (see Table S2 for mixed *dhps* genotypes) while 212 had single genotype at all 5 *dhps* codons based on single peaks in the sequencing electropherograms at each nucleotide. These 212 samples were subjected to neutral microsatellite (8 loci) analysis and 71 were found to contain multiple alleles at one or more neutral loci. Since it is not easy to construct unambiguous microsatellite haplotypes using the samples with multiple infections [26],[56], these 71 multiply-infected samples were excluded and only 141 single clonal samples were typed for microsatellites on chromosome 8 around the *dhps* gene. Therefore, we present here the *dhps* codon distribution for 212 isolates (Table 1) and *dhps* microsatellites data for 141 isolates.

**Table 1:** Prevalence of *dhps* mutations in Cambodia.

No. of mutations	DHPS alleles	Pailin n = 49	Kampong Seila n = 10	Chumkiri n = 80	Memut n = 37	Rattanakiri n = 36	Total n = 212 (%)
0	SAKAA	-	-	2	13	8	23 (10.8)
1	**A**AKAA	-	-	-	-	1	1 (0.47)
	S**G**KAA	-	3	3	2	13	21 (9.9)
2	**AG**KAA	-	-	2	7	7	16 (7.5)
	**FG**KAA	-	-	-	-	1	1 (0.47)
	S**GE**AA	3	-	7	12	2	24 (11.3)
	S**G**K**G**A	5	-	10	-	1	16 (7.5)
3	S**GEG**A	4	1	17	-	-	22 (10.3)
	S**GNG**A	22	2	16	1	-	41 (19.3)
	**AGE**AA	15	4	21	2	2	44 (20.7)
	**AG**KA**T**	-	-	-	-	1	1 (0.47)
4	**AGE**A**T**	-	-	1	-	-	1 (0.47)
	**FGE**A**T**	-	-	1	-	-	1 (0.47)

Note: The DHPS alleles are denoted by the single letter amino acid code for codons 436, 437, 540, 581 and 613. Mutated amino acids are boldfaced and underlined.

### A novel triple mutant *dhps* allele in Cambodia

Approximately 11% of the isolates, mostly from the east, had the ancestral wild type *dhps* allele S_436_A_437_K_540_A_581_A_613_ (SAKAA). None of the isolates from the west had the ancestral wild type allele (Table 1). We observed mutations at all five *dhps* codons implicated in sulfadoxine resistance, with striking differences in the regional distribution of *dhps* mutations in Cambodia. The *dhps* allele with the single 437G (SGKAA) mutation was seen in ∼10% of the isolates, mostly in the east. However, 437G alone or along with other *dhps* mutations were found in almost 89% of the isolates (Table 1). Collectively, the double mutant *dhps* alleles were found in nearly 27% of the isolates in either of three combinations, 436A/437G (AGKAA, 7.5% mostly in the east); 437G/581G (SGKGA, 7.5%, predominantly in the south and west); and 437G/540E (SGEAA, 11.3%, mostly in the south and east). Approximately 51% of the isolates harbored triple mutations of either 436A/437G/540E (AGEAA, 20.7%), 437G/540E/581G (SGEGA, 10.3%), the novel genotype 437G/540N/581G (SGNGA, 19.3%) or 436A/437G/613E (AGKAT, 0.47%). The alleles with triple mutations were predominant in western and southern Cambodia whereas the ancestral wild type, single, or double mutants were the predominant *dhps* alleles in the east (Table 1, Fig. 1). The finding of the novel triple mutant *dhps* allele SGNGA in high frequency (19.3%) is particularly interesting since this allele has not been reported from any other malaria endemic region of the world. The frequency of the SGNGA allele in Pailin was greater (∼45%) than the other highly resistant alleles. In addition, we found two quadruple mutant *dhps* alleles (AGEAT and FGEAT), albeit in low frequency (0.94%), in south Cambodia. We observed that 581G and 540E/N were always associated with 437G (SGKGA; SGEAA; SGEGA; AGEAA; SGNGA) (Table 1). Particularly, 540N was always associated with 437G and 581G.

### Genetic diversity and pattern of selective sweeps around *dhps* alleles

**Figure 2:**
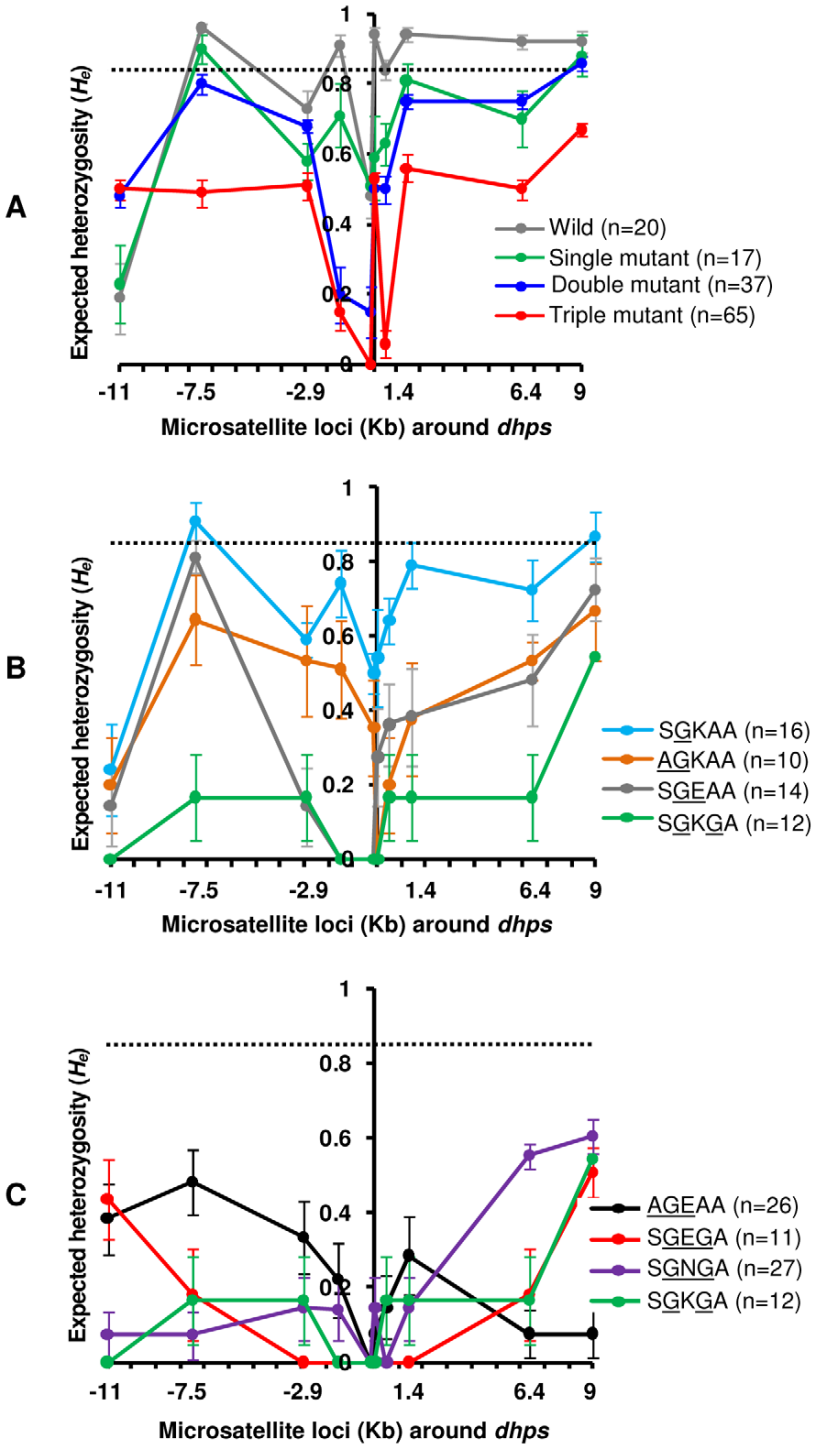
Selective sweeps around *dhps* alleles in Cambodia. (**A**) Comparison of the wild type and three mutant (single, double and triple) groups. (**B**) Comparison of the single (SGKAA) and double mutant (AGKAA, SGEAA, SGKGA) *dhps* alleles. (**C**) Comparison of the three triple mutant (AGEAA, SGEGA, SGNGA) *dhps* alleles. The dashed line crossing the y-axis indicates the mean heterozygosity (*H_e_*) at 8 neutral microsatellite loci on chromosomes 2 and 3. The error bars indicate ±1SD (standard deviation).

To analyze genetic diversity and investigate the pattern of selective sweeps around *dhps* alleles, we measured the number of alleles (A) and heterozygosity (*H_e_*) for each of the 10 loci. The mean number of alleles (A) as well as mean heterozygosity (*H_e_*) for the wild type *dhps* (SAKAA) were greater (A = 8.6±4.1; *H_e_* = 0.78±0.08) compared to *dhps* alleles harboring single (A = 5.9±3.2; *H_e_* = 0.65±0.06; *P* = 0.052), double (A = 4.5±2.4; *H_e_* = 0.56±0.07; *P* = 0.035), and triple mutations (A = 3.6±1.3; *H_e_* = 0.39±0.07; *P* = 0.005) (Fig. 2A, Fig. S1A; Table S3). This observation is compatible with these mutations being selected by drug pressure as indicated by a progressive decline in A and *H_e_* correlated with an increase in the number of favorable mutations in the *dhps* gene. Such a trend is consistent with a model of positive directional selection. To determine if the reduction in variation at *dhps* was possibly due to demographic effects, such as a population bottleneck, we also measured *H_e_* at 8 neutral loci on chromosomes 2 and 3 that are not known to be under any selection. The mean *H_e_* for the 8 neutral loci in Cambodia was significantly greater (*P* = 0.016) than the mean *H_e_* observed at 10 *dhps* loci. Moreover, there was no significant difference in *H_e_* at neutral loci when wild, single, double and triple mutant groups were compared (Table S3). Unlike selection which only acts on one or a few loci in a genome, demographic effects are expected to act on the entire genome, thus the decline in variation is not likely due to demographic effects [57]. As shown in Fig. 2A, the valley of reduced variation for the triple mutant *dhps* alleles was deeper followed by the double mutant and single mutant alleles, suggestive of stronger selection for the triple mutant alleles. With the exception of the −11 Kb locus, which generally exhibited lower diversity in both wild type and all mutant groups, there was sharp reduction in *H_e_* between −2.9 and 1.4 Kb region.

Since there were three types of double mutant *dhps* alleles in Cambodia, known to have different levels of *in vitro* resistance to sulfadoxine [13], we tested for differences in the selective sweeps around them. The double mutant SGKGA (A = 1.6±0.5; *H_e_* = 0.13±0.05) showed significantly lower diversity compared to AGKAA (A = 2.8±1.2; *H_e_* = 0.40±0.06; *P* = 0.01). However, there was no significant difference between the mean *H_e_* at SGEAA (A = 2.8±1.4; *H_e_* = 0.33±0.08) compared to SGKGA (*P* = 0.31) and AGKAA (*P* = 0.52) (Fig. 2B; Fig. S1B). The valley of reduced variation was also broader for SGKGA compared to the other two double mutants (Fig. 2B). All three triple mutant alleles (AGEAA: A = 2.7±1, *H_e_* = 0.20±0.05; SGNGA: A = 2.3±0.8, *H_e_* = 0.18±0.06; and SGEGA: A = 1.4±0.5, *H_e_* = 0.13±0.06) showed lower level of diversity with no significant difference between them (Fig. 2C; Fig. S1C). Interestingly, the valley was both wider and symmetrical for the SGEGA allele as compared to the SGNGA and AGEAA alleles (Fig. 2C). This could be the result of the expansion of few lineages with the SGEGA genotype because limited genetic variability within the founding population would broaden the width of the selective sweep. Alternatively, the SGEGA may have recently evolved with insufficient time for recombination to break down potential linkage. Although the mean *H_e_* for the loci surrounding SGKGA double mutant and SGEGA triple mutant alleles were almost similar (*P* = 0.48), there was some difference in the shape of the selection valley at these alleles (Fig. 2C).

### Comparison of genetic differentiation at *dhps* and neutral microsatellite loci

**Figure 3:**
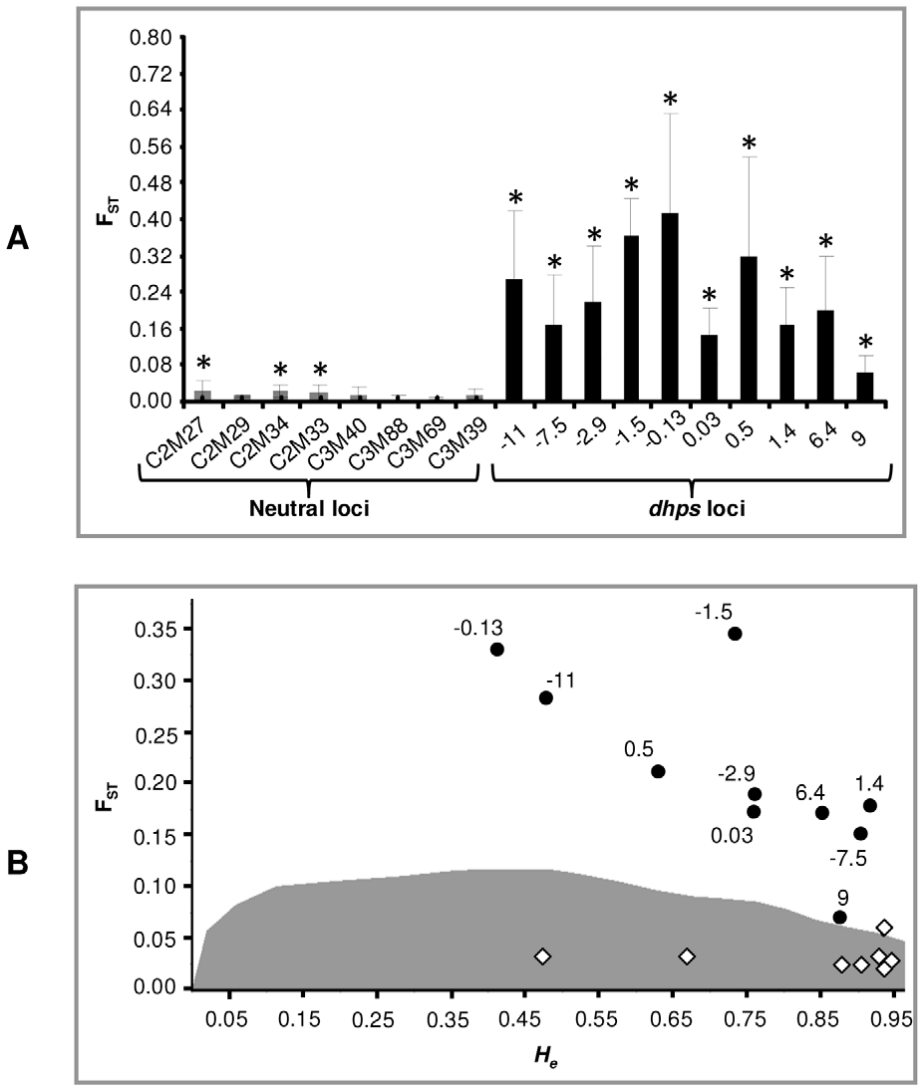
Comparison of F_ST_ across selected (*dhps*) and unselected (neutral) loci. (**A**) Histogram plotting F_ST_ (±SD) values at 8 neutral (gray bars) and 10 *dhps* (black bars) microsatellite loci. Asterisk indicates significant values obtained after 1000 random permutations. Populations were defined according to their *dhps* genotype (i.e. wild type, single, double, or triple mutant) for calculations of the overall (global) F_ST_. The overall F_ST_ for *dhps* after jackknifing over loci was 0.20 (95% CI: 0.15–0.26) whereas the overall F_ST_ for 8 neutral loci was 0.01 (95% CI: 0.006–0.019). The difference between F_ST_ values of *dhps* and neutral groups was highly significant as assessed by Mann-Whitney *U* test (Z = 3.55, *P* = 0.0003). (**B**) Plot of F_ST_ versus *H_e_* to disentangle selection on *dhps* and neutral loci. Black circles represent 10 *dhps* loci and white diamonds represent 8 neutral loci. The gray shading indicates the region of neutral expectations at a confidence level of 95%. The markers above the gray region are considered to be under directional selection while those inside the gray region are considered to be neutral. The *P* values (simulated F_ST_<sample F_ST_) for each locus are given as supporting information (Table S4).

The F_ST_ between populations (wild, single, double and triple mutant groups) were compared over 10 *dhps* and 8 neutral microsatellite loci as shown in Fig. 3A. As expected, F_ST_ values at all 10 *dhps* loci differed significantly from zero (Fig. 3A). On the other hand, three of the neutral loci also exhibited little but significant differentiation. The overall F_ST_ over the 10 *dhps* loci was 0.20 (SE = 0.03, CI = 0.15–0.26) and was significantly higher (Mann-Whitney *U* test: Z = 3.55, *P* = 0.0003) than the overall F_ST_ obtained over the 8 neutral loci (F_ST_ = 0.01, SE = 0.003 CI = 0.006–0.019).

The coalescent simulations method of Beaumont and Nichols [43] was used to disentangle selected loci from those behaving neutrally. The F_ST_ values for all *dhps* loci fell outside the 95% confidence interval of neutral expectations, indicative of being under directional selection or linked to a region of the genome under selection (Fig. 3B; see Table S4 for *P* value for deviation from neutral expectations). On the other hand, only one (C2M34) of the 8 neutral loci was found to lie marginally outside the 95% confidence interval (Fig. 3B; Table S4).

The pairwise F_ST_ comparisons between populations based on geography (4 regions in Cambodia) were performed using both *dhps* and neutral microsatellite loci (Table 2). The overall F_ST_ over the 10 *dhps* loci was 0.12, (SE = 0.02, CI = 0.09–0.16) and was significantly higher (Mann-Whitney *U* test: Z = −3.1, *P* = 0.0009) than the overall F_ST_ obtained over the 8 neutral loci (F_ST_ = 0.04, SE = 0.01, CI = 0.024–0.060).

**Table 2:** Pairwise F_ST_ comparisons between four populations in Cambodia.

	PL	CK	MM	RK
**PL**		**0.052**	0.017	**0.041**
**CK**	0.002		**0.045**	**0.059**
**MM**	**0.162**	**0.175**		0.011
**RK**	**0.160**	**0.180**	−0.005	

Note: Populations were defined according to geographic locations. Pailin: PL (n = 36); Chumkiri: CK (n = 50); Memut: MM, (n = 28); and Rattanakiri: RK (n = 27). Because there were only 7 singly-infected isolates from Kampong Seila these were combined with the PL population. F_ST_ values calculated from 10 *dhps* loci are given below the diagonal while those above are calculated from the 8 neutral loci. The values in bold indicate significant F_ST_ after Bonferroni correction (*P*≤0.008) for multiple comparisons. Overall F_ST_ value for *dhps* after jackknifing over loci was 0.12±0.02 (95% CI obtained after 1000 bootstrapping over loci: 0.09–0.16) and for neutral loci F_ST_ was 0.04±0.01 (95% CI: 0.024–0.060).

### Linkage disequilibrium (LD) around *dhps* alleles

A selective sweep reduces the amount of genetic variation at the chromosomal region containing a favorable mutation as well as at loci flanking the mutation, which leads to increased linkage disequilibrium (LD) around the target of selection. Therefore, in order to have an assessment of selection, we also measured LD as a standardized association index (I_A_^S^) between multiple loci flanking *dhps* alleles. There was no significant LD after Bonferroni correction (I_A_^S^ = 0.019, *P* = 0.06) around the wild type (SAKAA) allele, whereas significant LD was observed around mutant *dhps* alleles. The I_A_^S^ for single, double and triple mutant *dhps* alleles was 0.20 (*P*<0.01), 0.33 (*P*<0.01), and 0.22 (*P*<0.01), respectively.

### Multiple origins of resistant *dhps* alleles in Cambodia independent from Africa and South America

**Figure 4:**
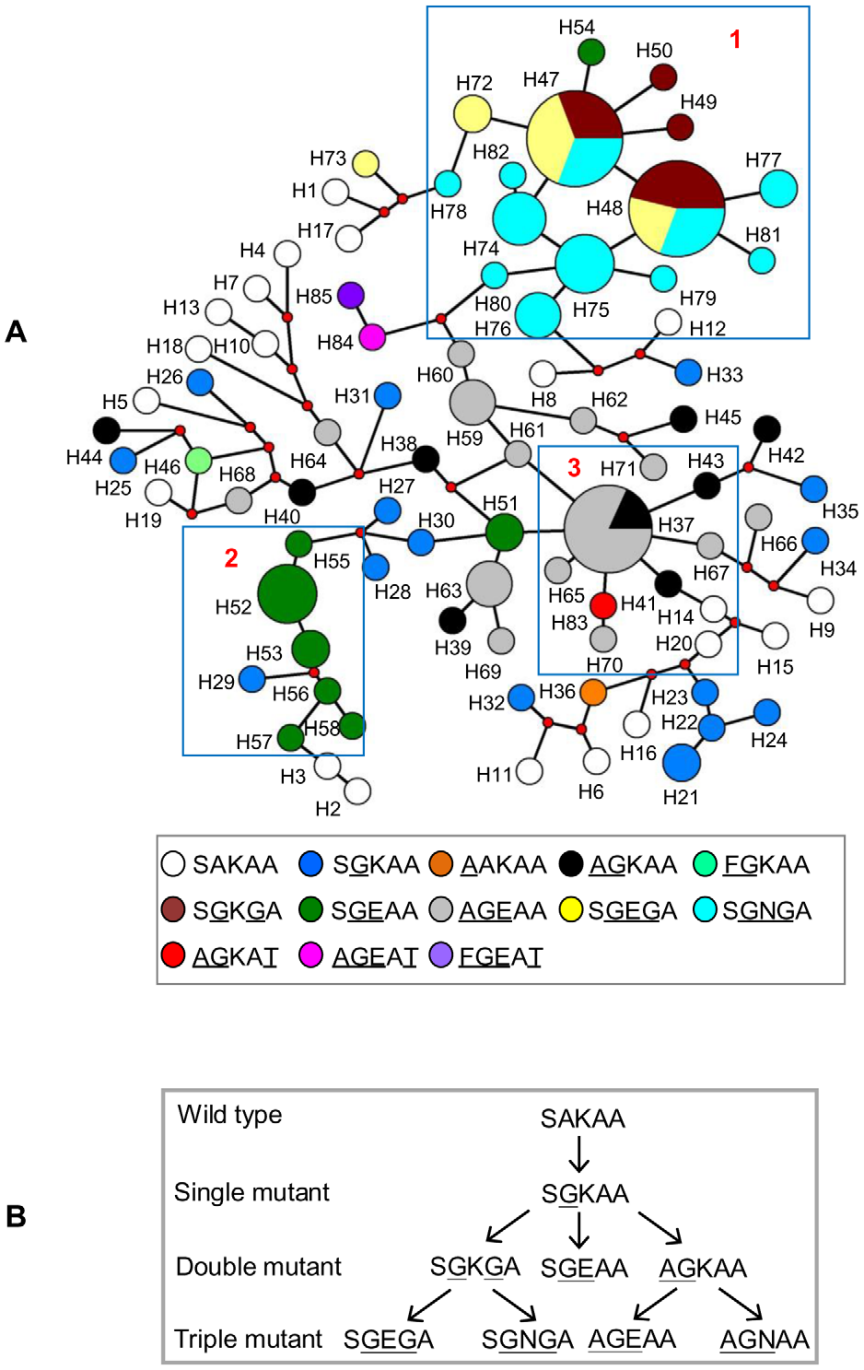
Genetic relationships among *dhps* alleles in Cambodia. (**A**) A median-joining network diagram depicting three independent origins for double mutant and two independent origins for triple mutant *dhps* alleles in Cambodia. The 141 *P. falciparum* isolates were classified into 85 haplotypes (H1 to H85; see individual haplotypes profiles in Table S5) based on the 10-loci microsatellite profile around *dhps* gene. The size of the circle is proportional to the number of isolates showing particular 10-loci microsatellite haplotype, the proportion of *dhps* alleles on that haplotype background are depicted by the pie charts. The red dots are hypothetical median vectors generated by the software to connect existing haplotypes within the network with maximum parsimony. Box 1, 2 and 3 indicate three major origins for *dhps* mutant alleles. (**B**) Scheme of plausible evolution of *dhps* alleles inferred from the microsatellite haplotypes data. Three major and independent lineages are proposed for the double mutants, one each for SGKGA, SGEAA and AGKAA. The SGKGA is the progenitor allele for SGEGA and SGNGA triple mutants, whereas AGKAA is the progenitor for AGEAA triple mutant. Most likely, the AGKAA double mutant could also be the progenitor of AGNAA triple mutant found on the Andaman and Nicobar Islands, given its wide occurrence on the islands.

We estimated the probable number of origin(s) for resistant *dhps* alleles in Cambodia based on the 10-loci microsatellite haplotypes around *dhps* (allele sizes and haplotypes information are given as supporting information in Table S5). The 10-loci haplotypes for all isolates with the wild type *dhps* allele were unique, showing complete linkage equilibrium between any two loci (open circles H1–H20 in Fig. 4A). The emergence of the 437G (SGKAA) mutation on multiple wild type genetic backgrounds led to an increase in the LD between loci and limited sharing of microsatellite haplotypes between the isolates (blue circles, particularly H21 to H24 in Fig. 4A). We found three major independent lineages for the double mutant *dhps* alleles: SGKGA, SGEAA and AGKAA in Cambodia (Fig. 4A). This was evident from the lack of shared 10-loci microsatellite haplotypes between these three alleles (Table S5). The SGKGA alleles shared identical microsatellite haplotype backgrounds, suggesting a single origin for this allele (maroon circles labeled H47 to H50 in Fig. 4A). Although a majority of the SGEAA allele shared common microsatellite haplotypes, a minority were also found to occur on diverse backgrounds (green circles in Fig. 4A). A similar scenario was also observed for the AGKAA allele (black circles in Fig. 4A). Among the triple mutants, the SGEGA and SGNGA alleles shared a common origin and appear to have emerged from the SGKGA double mutant, as indicated by their common haplotype backgrounds (Fig. 4A, 4B; Table S5). On the other hand, the AGEAA triple mutant originated from AGKAA on identical or nearly identical genetic backgrounds, suggesting multiple origins for this allele or possibly recombination events (Fig. 4A, 4B, Table S5). We did not find any evidence suggestive of the emergence of any triple mutant *dhps* alleles resulting from the SGEAA genetic background (Fig. 4A, 4B). Thus, our results seem to suggest multiple but limited origins for the highly resistant *dhps* alleles in Cambodia.

**Figure 5:**
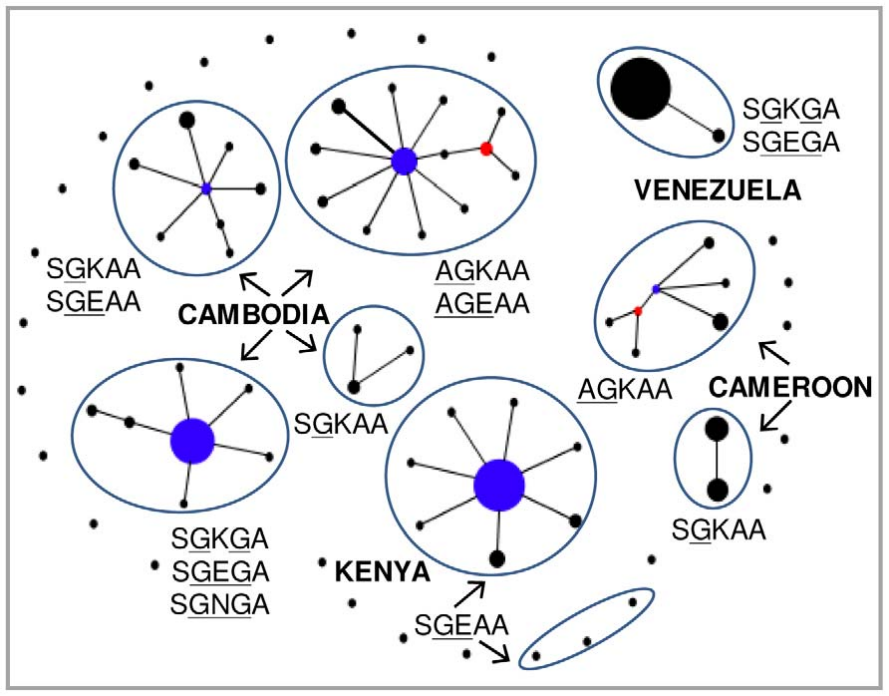
Genetic relationships among the *dhps* alleles of Southeast Asia (Cambodia), Africa (Kenya and Cameroon) and South America (Venezuela). An eBURST diagram showing major lineages for Cambodian *dhps* alleles. Two major lineages exist in Cameroon (SGKAA and AGKAA), one major lineage in Kenya (SGEAA), and one in Venezuela (SGKGA and SGEGA). Each lineage is distinct from the others, suggesting multiple, independent evolutionary histories.

We compared the 7-loci microsatellite haplotype of the Cambodian *dhps* alleles with the Kenyan (SGEAA) and Cameroonian (SGKAA and AGKAA) *dhps* alleles (Fig. 5). In addition, we also compared backgrounds of the Cambodian alleles with the SGKGA and SGEGA alleles from Venezuela. Within Kenya, the majority of the SGEAA alleles had identical or nearly identical 7-loci microsatellite haplotypes (Fig. 5). However, a few SGEAA alleles also had unique haplotypes suggesting independent evolution of these alleles or recombination events. None of the isolates in Kenya and Cambodia shared identical 7-loci microsatellite haplotypes, suggesting local evolution of SGEAA allele in Kenya independent from Cambodia (Fig. 5). Similarly, unique haplotype profiles of the SGKAA and AGKAA alleles in Cameroon suggest multiple origins independent from Cambodia (Fig. 5). The only triple mutant found in South America (with a common founder) is SGEGA, and this allele had a completely distinct origin from the Cambodian SGKGA and SGEGA alleles. The double mutant SGKGA allele in Venezuela also had a completely distinct microsatellite haplotype compared to the Cambodian counterparts, indicating an independent evolution of *dhps* mutants in the South American region (Fig. 5).

## Discussion

Unlike CQ and pyrimethamine resistance, there is no information available on the origins and evolutionary dynamics of sulfadoxine-resistant *dhp*s alleles in the *P. falciparum* population from Southeast Asia. Therefore, the present study was undertaken to reveal the lineages of the highly resistant *dhps* alleles in this region. Samples in this study were analyzed from Pailin on the Thailand-Cambodia border as well as from four other regions in eastern and southern Cambodia. The isolates from the east predominantly had wild type (SAKAA), single mutant (SGKAA), and double mutant (AGKAA and SGEAA) *dhps* alleles, whereas those from the west and south predominantly had SGKGA double mutant and SGEGA, SGNGA and AGEAA triple mutants. These findings are in accordance with a previous finding that the parasites from eastern Cambodia are generally less resistant to SP (Cambodia National Malaria Control Program, unpublished data). It is important to note that although SP officially has not been in use in Cambodia for more than a decade, sulfadoxine-resistant *dhps* alleles are rampant in the population. This may also be explained by the easy availability of SP as a cheap over-the-counter antimalarial in Cambodia, particularly in rural areas. Interestingly, almost all mutant *dhps* alleles (188/189) had the 437G mutation, consistent with its critical role in sulfadoxine resistance and its wide occurrence globally [24],[58],[59]. The first mutation to occur and persist in populations in response to sulfadoxine pressure is 437G followed by mutations at additional codons, which lead to progressive increases in the level of resistance [15]. The 437G (SGKAA) or 437G/540E (SGEAA) *dhps* alleles in conjunction with double (51I//59R) or triple (51I/59R/108N) mutant *dhfr* alleles have been found to be associated with SP therapeutic failure in studies from Africa and Asia [58],[59],[60],[61],[62],[63],[64],[65],[66],[67],[68]. Similarly, the 437G/540E/581G (SGEGA) allele with the *dhfr* 51I//108N/164L allele has been correlated with *in vivo* SP resistance in South America [19].

Approximately 20% of the isolates, mainly from the west and south Cambodia, harbored a 540N mutation, always in association with 437G/581G (SGNGA). The Pailin area had ∼45% isolates with this novel triple mutant *dhps* allele (Table 1). To our knowledge, this is the first time that this allele has been observed in any malaria-endemic region of the world. However, it is not known how and when this new mutation emerged and what role this mutation plays in the response of the sulfadoxine. Nonetheless, its mere occurrence on the Thailand-Cambodia border and in southern Cambodia and strong selective signature around this allele (Fig. 2), suggest that this mutation may be playing an important role in sulfadoxine resistance. It is also likely that cross resistance to related drugs like cotrimoxazole (trimethoprim plus sulfamethoxazole), may have a role in selection of this allele. Cotrimoxazole is another antifolate combination widely used to treat bacterial infections. In one study, *dhps* mutations were analyzed in 53 Cambodian isolates, however the 540N mutation was not found, possibly due to the fact that most of these samples were from the eastern part of the country [16]. Similarly, we found only one isolate with the 540N mutation in eastern Cambodia (Table 1). However, a recent study has reported 540N *dhps* mutation in 19 of the 55 *P. falciparum* isolates from the Car Nicobar Islands of India, collected in October 2005 almost 9 months after the December 2004 tsunami, though; it was seen in association with 436A/437G (AGNAA) [69], unlike what we found in Cambodia. The 540N mutation was not found in samples (n = 50) collected before the tsunami. Lumb et al. [69] argue that after the tsunami many patients were simultaneously treated with two antifolate combinations: SP for malaria and trimethoprim-sulfamethoxazole for bacterial infections. This strong antifolate pressure may have allowed the selection of 540N mutation in the population. Interestingly, this mutation was not seen in samples collected at four other time points after the tsunami (n = 103) [69]. Like SGNGA, the role of the AGNAA *dhps* allele in sulfadoxine resistance is yet to be determined.

As expected under a model of a selective sweep, the *dhps* alleles with three mutations had the highest reduction in diversity at their flanking loci, followed by double and single mutant alleles (Fig. 2A). We also noted that the pattern of selective sweep at each allele was broadly correlated with the level of *in vitro* resistance to sulfadoxine. The triple mutant allele AGEAA had greater reduction in heterozygosity (inhibitory constant for sulfadoxine, *K_i_* = 98.3±7.5 µM) followed by SGKGA (*K_i_* = 16.2±2.7 µM), SGEAA (*K_i_* = 27.7±6.1 µM), AGKAA (*K_i_* = 19.9±4.0 µM), SGKAA (*K_i_* = 1.39±0.23 µM) and SAKAA (wild type, *K_i_* = 0.14±0.01 µM) [13]. The reduction in heterozygosity due to a selective sweep was greatest for the SGEGA and SGNGA triple mutant alleles indicating stronger selection for these alleles (Fig. 2C). However, the *K_i_*'s of these alleles for sulfadoxine are not known.

Underlying population structure may influence the amount of gene flow between resistant parasites, clouding estimates of selection. However, we have shown there are few barriers to gene flow either based on mutation or geography using the neutral loci (Fig. 3A and Table 2). Our estimates of global F_ST_ using the neutral microsatellite loci are below 0.05 using either geography or mutation to partition variation in Cambodia, indicating little genetic structuring in this population of *P. falciparum*. In contrast, quite high estimates of F_ST_ (greater than 0.12) are obtained using the microsatellite loci surrounding *dhps* (Fig. 3A) clearly supporting a role for strong selective pressure on this gene. Further, the F_ST_ outlier analysis also suggest that all 10 *dhps* loci have statistically higher F_ST_ values than expected under neutrality (Fig. 3B; Table S4), supporting that these loci are subject to directional selection.

The present data suggest three major independent origins of double mutant *dhps* alleles in Cambodia. The 437G mutation (SGKAA) was found to occur on multiple genetic backgrounds with subsequent mutations at 540, 436 and 581 producing the double mutants SGEAA, AGKAA and SGKGA, respectively (Fig. 4B). In accordance, the microsatellite haplotype backgrounds of these three double mutants were distinct from each other (Panel C, D and E in Table S5). Further, the SGKGA double mutant after acquiring 540E or 540N gave rise to SGEGA or SGNGA triple mutants, respectively, which is strongly supported by their common microsatellite backgrounds (Fig. 4B, Panel C of Table S5). Thus, SGEGA and SGNGA alleles have a common origin from the double mutant SGKGA. The SGKGA and SGEGA alleles in South America also have been found to share single and common haplotype backgrounds [21] yet different from Cambodia. At this point, we do not know whether 540E and 540N mutation in Cambodia simultaneously emerged and rose in frequency under sulfadoxine pressure or if they emerged at different time points. Analysis of the retrospective samples from this region may provide some clue on this aspect. However, because the 540E mutation is so widespread, it is likely that this would have been the first mutation to occur followed by 540N. Similarly, the widely spread triple mutant *dhps* allele AGEAA in Asia [17],[18] likely resulted from the AGKAA double mutant acquiring the mutation at codon 540. As shown in Fig. 4A (also in panel D of Table S5), the double and triple mutants share identical haplotype backgrounds, reaffirming their shared origin. It is likely that the novel AGNAA triple mutant allele observed on the Car Nicobar Islands [69] may have also emerged on the AGKAA background, similar to AGEAA (Fig. 4B). Thus, our data suggest that there are three major independent origins for the double mutant *dhps* alleles and two major independent origins for the triple mutant alleles in Cambodia (Fig. 4).

Gene flow plays an important role in the spread of drug resistant mutations as has been seen in the case of CQ and pyrimethamine resistance. Genetic evidence suggests that CQ and pyrimethamine resistance originated on the Thailand-Cambodia border and spread to Africa in the late 1970s and mid 1980s, respectively [26],[31],[70]. Thus, we also attempted to understand the evolutionary relationships between the Southeast Asian, African and South American *dhps* alleles. Results suggest that the SGEGA alleles both in South America [20],[21] and Cambodia evolved independently (Fig. 5). In a recent study, Pearce et al. [24] attempted to understand the origin and dispersal of sulfadoxine resistance in Africa by analyzing three microsatellite loci flanking the *dhps* gene. Based on the data from several African countries it has been concluded that the sulfadoxine resistance-conferring *dhps* alleles in east Africa (predominantly SGEAA) are different from those in west Africa (SGKAA and AGKAA) and these alleles are associated with multiple genetic backgrounds consisting of five major lineages [24]. This corroborates with our previous data [25] as well as the present study, which confirms multiple, independent origins of the above *dhps* alleles in east and west Africa (Fig. 5). None of the triple mutant *dhps* alleles reported in other parts of the world has appeared in Africa. It remains to be seen whether a triple mutant *dhps* allele will independently evolve in Africa or expand from migration of parasites from other parts of the world, as in the case of triple mutant *dhfr*. It is surprising though that the major triple mutant *dhfr* allele found in Africa is believed to have been imported from Southeast Asia and spread across the continent while none of the triple mutant *dhps* alleles have been established in Africa. One possibility may be that the introduction of *dhfr* mutant allele may have preceded the development of triple mutant *dhps* allele.

In conclusion, results from this study reveal some very interesting findings: i) presence of a novel triple mutant *dhps* SGNGA as a predominant allele in Cambodia along with two previously reported triple mutant alleles SGEGA and AGEAA; ii) the triple mutant *dhps* alleles were present at high frequencies in the western and southern parts of Cambodia while they were rarely seen in the east, indicating that sulfadoxine resistance may be a lesser problem in the east; iii) the triple mutant *dhps* alleles have not declined over several years after SP was officially removed as the drug of choice for the primary treatment of malaria in this region; iv) at least three independent origins of the double mutants and two independent origins of triple mutant *dhps* alleles are evident in Cambodia; v) the origin of triple mutant *dhps* allele SGEGA in South America is completely distinct from the Cambodian SGEGA allele; vi) the double mutant SGEAA allele in Kenya and AGKAA allele in Cameroon are evolutionary distinct from their counterparts in Cambodia, indicating multiple origins for these alleles. Finally, it is also evident that unlike *pfcrt* and *dhfr* resistant alleles, which have single origin in Thailand-Cambodia region, the *dhps* resistant alleles have multiple origins in this region. Thus, we have provided comprehensive new data illustrating multiple global origins for sulfadoxine resistant *dhps* alleles.

## Supporting Information

Table S1 List of primers used to amplify 10 microsatellite loci around *dhps* gene
(0.03 MB DOC)

Table S2 Distribution of the DHPS genotypes in the 22 multiply-infected *P. falciparum* samples
(0.05 MB DOC)

Table S3 The expected heterozygosity (*H*_e_) and number of alleles (A) at 10 microsatellite loci around *dhps* gene and at 8 loci on chromosomes 2 and 3 in Cambodia.
(0.07 MB DOC)

Table S4 Sample *H*_e_ and sample F_ST_ at all 10 *dhps* and 8 neutral microsatellite loci as obtained in LOSITAN
(0.04 MB DOC)

Table S5 The 10-loci microsatellite haplotype profiles of the *dhps* alleles in Cambodia
(0.04 MB PDF)

Figure S1 Box-and-Whisker plot comparing the heterozygosities (*H*_e_) around *dhps* alleles in Cambodia. The box indicates the median (horizontal solid line inside the box) and interquartile range (25th and 75th percentiles) and the whiskers indicate the range (upper and lower *H*_e_ values). The outliers are shown as solid black diamonds. The difference between each group was compared using Mann-Whitney *U* test and *P* values≤0.05 were considered significant. Statistically significant differences are indicated above the box by *P* values. (A) *H*_e_ comparison between wild (n = 20), single (n = 17), double (n = 37) and triple (n = 65) mutant dhps alleles. (B) *H*_e_ comparison between SGKAA (n = 16), AGKAA (n = 10), SGEAA (n = 14) and SGKGA (n = 12) *dhps* alleles. (C) *H*_e_ comparison between SGKGA (n = 12), AGEAA (n = 26), SGNGA (n = 27), SGEGA (n = 11) *dhps* alleles. Similar analyses were also done for all neutral microsatellite loci, however none of the comparison produced statistically significant difference.
(0.94 MB TIF)

## References

[1] Eyles DE, Hoo CC, Warren M, Sandosham AA (1963). Plasmodium falciparum resistant to chloroquine in Cambodia.. Am J Trop Med Hyg.

[2] Verdrager J (1986). Epidemiology of the emergence and spread of drug-resistant falciparum malaria in South-East Asia and Australasia.. J Trop Med Hyg.

[3] Hurwitz ES, Johnson D, Campbell CC (1981). Resistance of Plasmodium falciparum malaria to sulfadoxine-pyrimethamine (‘Fansidar’) in a refugee camp in Thailand.. Lancet.

[4] Wongsrichanalai C, Meshnick SR (2008). Declining artesunate-mefloquine efficacy against falciparum malaria on the Cambodia-Thailand border.. Emerg Infect Dis.

[5] WHO (2002). Development of South-Asia surveillance network for malaria drug resistance..

[6] Hurly MG (1959). Potentiation of pyrimethamine by sulphadiazine in human malaria.. Trans R Soc Trop Med Hyg.

[7] Chulay JD, Watkins WM, Sixsmith DG (1984). Synergistic antimalarial activity of pyrimethamine and sulfadoxine against Plasmodium falciparum in vitro.. Am J Trop Med Hyg.

[8] Laing AB (1970). Studies on the chemotherapy of malaria. I. The treatment of overt falciparum malaria with potentiating combinations of pyrimethamine and sulphormethoxine or dapsone in The Gambia.. Trans R Soc Trop Med Hyg.

[9] WHO (2008).

[10] Brown GM (1962). The biosynthesis of folic acid. II. Inhibition by sulfonamides.. J Biol Chem.

[11] Brooks DR, Wang P, Read M, Watkins WM, Sims PF (1994). Sequence variation of the hydroxymethyldihydropterin pyrophosphokinase: dihydropteroate synthase gene in lines of the human malaria parasite, Plasmodium falciparum, with differing resistance to sulfadoxine.. Eur J Biochem.

[12] Triglia T, Cowman AF (1994). Primary structure and expression of the dihydropteroate synthetase gene of Plasmodium falciparum.. Proc Natl Acad Sci U S A.

[13] Triglia T, Menting JG, Wilson C, Cowman AF (1997). Mutations in dihydropteroate synthase are responsible for sulfone and sulfonamide resistance in Plasmodium falciparum.. Proc Natl Acad Sci U S A.

[14] Wang P, Read M, Sims PF, Hyde JE (1997). Sulfadoxine resistance in the human malaria parasite Plasmodium falciparum is determined by mutations in dihydropteroate synthetase and an additional factor associated with folate utilization.. Mol Microbiol.

[15] Triglia T, Wang P, Sims PF, Hyde JE, Cowman AF (1998). Allelic exchange at the endogenous genomic locus in Plasmodium falciparum proves the role of dihydropteroate synthase in sulfadoxine-resistant malaria.. EMBO J.

[16] Khim N, Bouchier C, Ekala MT, Incardona S, Lim P (2005). Countrywide survey shows very high prevalence of Plasmodium falciparum multilocus resistance genotypes in Cambodia.. Antimicrob Agents Chemother.

[17] van den Broek IV, van der Wardt S, Talukder L, Chakma S, Brockman A (2004). Drug resistance in Plasmodium falciparum from the Chittagong Hill Tracts, Bangladesh.. Trop Med Int Health.

[18] Ahmed A, Lumb V, Das MK, Dev V, Wajihullah (2006). Prevalence of mutations associated with higher levels of sulfadoxine-pyrimethamine resistance in Plasmodium falciparum isolates from Car Nicobar Island and Assam, India.. Antimicrob Agents Chemother.

[19] Kublin JG, Witzig RS, Shankar AH, Zurita JQ, Gilman RH (1998). Molecular assays for surveillance of antifolate-resistant malaria.. Lancet.

[20] Cortese JF, Caraballo A, Contreras CE, Plowe CV (2002). Origin and dissemination of Plasmodium falciparum drug-resistance mutations in South America.. J Infect Dis.

[21] McCollum AM, Mueller K, Villegas L, Udhayakumar V, Escalante AA (2007). Common origin and fixation of Plasmodium falciparum dhfr and dhps mutations associated with sulfadoxine-pyrimethamine resistance in a low-transmission area in South America.. Antimicrob Agents Chemother.

[22] Bacon DJ, McCollum AM, Griffing SM, Salas C, Soberon V (2009). Dynamics of malaria drug resistance patterns in the Amazon basin region following changes in Peruvian national treatment policy for uncomplicated malaria.. Antimicrob Agents Chemother.

[23] Zhou Z, Griffing SM, de Oliveira AM, McCollum AM, Quezada WM (2008). Decline in sulfadoxine-pyrimethamine-resistant alleles after change in drug policy in the Amazon region of Peru.. Antimicrob Agents Chemother.

[24] Pearce RJ, Pota H, Evehe MS, Ba el H, Mombo-Ngoma G (2009). Multiple origins and regional dispersal of resistant dhps in African Plasmodium falciparum malaria.. PLoS Med.

[25] McCollum AM, Basco LK, Tahar R, Udhayakumar V, Escalante AA (2008). Hitchhiking and selective sweeps of Plasmodium falciparum sulfadoxine and pyrimethamine resistance alleles in a population from central Africa.. Antimicrob Agents Chemother.

[26] Nair S, Williams JT, Brockman A, Paiphun L, Mayxay M (2003). A selective sweep driven by pyrimethamine treatment in southeast asian malaria parasites.. Mol Biol Evol.

[27] McCollum AM, Poe AC, Hamel M, Huber C, Zhou Z (2006). Antifolate resistance in Plasmodium falciparum: multiple origins and identification of novel dhfr alleles.. J Infect Dis.

[28] Mita T, Tanabe K, Takahashi N, Culleton R, Ndounga M (2009). Indigenous evolution of Plasmodium falciparum pyrimethamine resistance multiple times in Africa.. J Antimicrob Chemother.

[29] Certain LK, Briceno M, Kiara SM, Nzila AM, Watkins WM (2008). Characteristics of Plasmodium falciparum dhfr haplotypes that confer pyrimethamine resistance, Kilifi, Kenya, 1987–2006.. J Infect Dis.

[30] Roper C, Pearce R, Bredenkamp B, Gumede J, Drakeley C (2003). Antifolate antimalarial resistance in southeast Africa: a population-based analysis.. Lancet.

[31] Roper C, Pearce R, Nair S, Sharp B, Nosten F (2004). Intercontinental spread of pyrimethamine-resistant malaria.. Science.

[32] Maiga O, Djimde AA, Hubert V, Renard E, Aubouy A (2007). A shared Asian origin of the triple-mutant dhfr allele in Plasmodium falciparum from sites across Africa.. J Infect Dis.

[33] Ariey F, Fandeur T, Durand R, Randrianarivelojosia M, Jambou R (2006). Invasion of Africa by a single pfcrt allele of South East Asian type.. Malar J.

[34] Ndiaye D, Daily JP, Sarr O, Ndir O, Gaye O (2006). Defining the origin of Plasmodium falciparum resistant dhfr isolates in Senegal.. Acta Trop.

[35] Lynch C, Pearce R, Pota H, Cox J, Abeku TA (2008). Emergence of a dhfr mutation conferring high-level drug resistance in Plasmodium falciparum populations from southwest Uganda.. J Infect Dis.

[36] Mita T, Tanabe K, Takahashi N, Tsukahara T, Eto H (2007). Independent evolution of pyrimethamine resistance in Plasmodium falciparum isolates in Melanesia.. Antimicrob Agents Chemother.

[37] McCollum AM (2007).

[38] Su X, Ferdig MT, Huang Y, Huynh CQ, Liu A (1999). A genetic map and recombination parameters of the human malaria parasite Plasmodium falciparum.. Science.

[39] Peakall R, Smouse PE (2006). GENALEX 6: genetic analysis in Excel. Population genetic software for teaching and research.. Mol Ecol Notes.

[40] Weir BS, Cockerham CC (1984). Estimating F-statistics for the analysis of population structure.. Evolution.

[41] Lewis PO, Zaykin D (2001). Genetic Data Analysis: Computer program for the analysis of allelic data. Version 1.0 (d16c).. http://lewis.eeb.uconn.edu/lewishome/software.html.

[42] Lewontin RC, Krakauer J (1973). Distribution of gene frequency as a test of the theory of the selective neutrality of polymorphisms.. Genetics.

[43] Beaumont MA, Nichols RA (1996). Evaluating loci for use in the genetic analysis of population structure.. Proc R Soc Lond B: Biol Sci.

[44] Wilding CS, Butlin RK, Grahame J (2001). Differential gene exchange between parapatric morphs of *Littorina saxatilis* detected using AFLP markers.. J Evol Biol.

[45] Anderson TJ, Nair S, Sudimack D, Williams JT, Mayxay M (2005). Geographical distribution of selected and putatively neutral SNPs in Southeast Asian malaria parasites.. Mol Biol Evol.

[46] Storz JF (2005). Using genome scans of DNA polymorphism to infer adaptive population divergence.. Mol Ecol.

[47] Beaumont MA (2005). Adaptation and speciation: what can F(st) tell us?. Trends Ecol Evol.

[48] Demontis D, Pertoldi C, Loeschcke V, Mikkelsen K, Axelsson T (2009). Efficiency of selection, as measured by single nucleotide polymorphism variation, is dependent on inbreeding rate in Drosophila melanogaster.. Mol Ecol.

[49] Antao T, Lopes A, Lopes RJ, Beja-Pereira A, Luikart G (2008). LOSITAN: a workbench to detect molecular adaptation based on a Fst-outlier method.. BMC Bioinformatics.

[50] Goudet J (2001).

[51] Smith JM, Smith NH, O'Rourke M, Spratt BG (1993). How clonal are bacteria?. Proc Natl Acad Sci U S A.

[52] Hudson RR (1994). Analytical results concerning linkage disequilibrium in models with genetic transformation and conjugation.. J Evol Biol.

[53] Haubold B, Hudson RR (2000). LIAN 3.0: detecting linkage disequilibrium in multilocus data. Linkage Analysis.. Bioinformatics.

[54] Bandelt HJ, Forster P, Rohl A (1999). Median-joining networks for inferring intraspecific phylogenies.. Mol Biol Evol.

[55] Feil EJ, Li BC, Aanensen DM, Hanage WP, Spratt BG (2004). eBURST: inferring patterns of evolutionary descent among clusters of related bacterial genotypes from multilocus sequence typing data.. J Bacteriol.

[56] Nash D, Nair S, Mayxay M, Newton PN, Guthmann JP (2005). Selection strength and hitchhiking around two anti-malarial resistance genes.. Proc Biol Sci.

[57] Harris EE, Hey J (1999). X chromosome evidence for ancient human histories.. Proc Natl Acad Sci U S A.

[58] Gregson A, Plowe CV (2005). Mechanisms of resistance of malaria parasites to antifolates.. Pharmacol Rev.

[59] Picot S, Olliaro P, de Monbrison F, Bienvenu AL, Price RN (2009). A systematic review and meta-analysis of evidence for correlation between molecular markers of parasite resistance and treatment outcome in falciparum malaria.. Malar J.

[60] Curtis J, Duraisingh MT, Warhurst DC (1998). In vivo selection for a specific genotype of dihydropteroate synthetase of Plasmodium falciparum by pyrimethamine-sulfadoxine but not chlorproguanil-dapsone treatment.. J Infect Dis.

[61] Wang P, Lee CS, Bayoumi R, Djimde A, Doumbo O (1997). Resistance to antifolates in Plasmodium falciparum monitored by sequence analysis of dihydropteroate synthetase and dihydrofolate reductase alleles in a large number of field samples of diverse origins.. Mol Biochem Parasitol.

[62] Nzila AM, Mberu EK, Sulo J, Dayo H, Winstanley PA (2000). Towards an understanding of the mechanism of pyrimethamine-sulfadoxine resistance in Plasmodium falciparum: genotyping of dihydrofolate reductase and dihydropteroate synthase of Kenyan parasites.. Antimicrob Agents Chemother.

[63] Omar SA, Adagu IS, Gump DW, Ndaru NP, Warhurst DC (2001). Plasmodium falciparum in Kenya: high prevalence of drug-resistance-associated polymorphisms in hospital admissions with severe malaria in an epidemic area.. Ann Trop Med Parasitol.

[64] Staedke SG, Sendagire H, Lamola S, Kamya MR, Dorsey G (2004). Relationship between age, molecular markers, and response to sulphadoxine-pyrimethamine treatment in Kampala, Uganda.. Trop Med Int Health.

[65] Kun JF, Lehman LG, Lell B, Schmidt-Ott R, Kremsner PG (1999). Low-dose treatment with sulfadoxine-pyrimethamine combinations selects for drug-resistant Plasmodium falciparum strains.. Antimicrob Agents Chemother.

[66] Kublin JG, Dzinjalamala FK, Kamwendo DD, Malkin EM, Cortese JF (2002). Molecular markers for failure of sulfadoxine-pyrimethamine and chlorproguanil-dapsone treatment of Plasmodium falciparum malaria.. J Infect Dis.

[67] Kyabayinze D, Cattamanchi A, Kamya MR, Rosenthal PJ, Dorsey G (2003). Validation of a simplified method for using molecular markers to predict sulfadoxine-pyrimethamine treatment failure in African children with falciparum malaria.. Am J Trop Med Hyg.

[68] Nagesha HS, Din S, Casey GJ, Susanti AI, Fryauff DJ (2001). Mutations in the pfmdr1, dhfr and dhps genes of Plasmodium falciparum are associated with in-vivo drug resistance in West Papua, Indonesia.. Trans R Soc Trop Med Hyg.

[69] Lumb V, Das MK, Mittra P, Ahmed A, Kumar M (2009). Emergence of an unusual sulfadoxine-pyrimethamine resistance pattern and a novel K540N mutation in dihydropteroate synthetase in Plasmodium falciparum isolates obtained from Car Nicobar Island, India, after the 2004 Tsunami.. J Infect Dis.

[70] Wootton JC, Feng X, Ferdig MT, Cooper RA, Mu J (2002). Genetic diversity and chloroquine selective sweeps in Plasmodium falciparum.. Nature.

