# Supplementary material for: Origin and Evolution of Sulfadoxine Resistant Plasmodium falciparum
Source: PLoS Pathog. 2010 Mar 26;6(3):e1000830. doi: 10.1371/journal.ppat.1000830 (PMC2847944; doi:10.1371/journal.ppat.1000830)
Supplement: Table S1 — List of primers used to amplify 10 microsatellite loci around dhps gene (0.03 MB DOC) [file ppat.1000830.s001.doc]

**Table S1:** List of primers used to amplify 10 microsatellite loci around *dhps* gene.

| **Loci (Kb)** | **Forward (5’-3’)** | **Reverse (5’-3’)** | **PlasmoDB position (bp)^#^** |
| --- | --- | --- | --- |
| -11 | AACTTATACGTATCTAAAG | TGCGGGTATAATACATTA* | 538108-538328 |
| -7.5 | TTTTAACTTGTATCAAGAAAT | TACAGCACTTAATGTAAATGGAG* | 541762-541928 |
| -2.9 | ATGTTTGAACCCCTTAATTTA | CACATGTAAATGCATATTTATG* | 546336-546530 |
| -1.5 | TGTCTTGAAGGACAACACATAGATG | CATAATATGAAGAGACTGAAAGTT* | 547785-547982 |
| -0.13 | AAATATTTGCGCCAAACTTT | TAGATTTCTTTACGCAAAAT* | 549133-549270 |
| ***dhps*** |  |  |  |
| 0.03 | GGAAAGTGCAAACATGT* | AGAGTACTTGACATATAATGAGCATG | 551747-551898 |
| 0.5 | AGGAAAGTGTACGACGTTTATTGAATG | AGGACTGATCATATTACCAAG* | 552177-552327 |
| 1.4 | GCATTCACACCAGTCTGCCTTCAA | AGGAGGTTTCCCTTCACTCCATCT* | 553007-553254 |
| 6.4 | AGCTTTTCTCCGACAAACCAAG | CCTGAAAAGTGAGGAATGCG* | 557940-558240 |
| 9 | TGGAATTCATATTAATTGTAC | GAATAAATTAATTACACACGGAA* | 560710-560825 |

*; FAM/HEX labeled primer

**#**; Indicate position of forward and reverse primers on chromosome 8 of 3D7 ([http://plasmodb.org](http://plasmodb.org/)).

*Note:* The PCR cycling parameters for all primers were as follows: Initial denaturation at 94°C for 2 minutes, followed by 5 cycles of 94°C for 30 seconds, 50°C for 30 seconds, 60°C for 30 seconds; and 25 cycles of 94°C for 30 seconds, 45°C for 30 seconds, 60°C for 30 seconds.
