# Supplementary material for: Origin and Evolution of Sulfadoxine Resistant Plasmodium falciparum
Source: PLoS Pathog. 2010 Mar 26;6(3):e1000830. doi: 10.1371/journal.ppat.1000830 (PMC2847944; doi:10.1371/journal.ppat.1000830)
Supplement: Table S2 — Distribution of the DHPS genotypes in the 22 multiply-infected P. falciparum samples (0.05 MB DOC) [file ppat.1000830.s002.doc]

**Table S2:** Distribution of the DHPS genotypes in the 22 multiply-infected *P. falciparum* samples

| **DHPS codons** | | | | | **Regions** | | | | |
| --- | --- | --- | --- | --- | --- | --- | --- | --- | --- |
| **436** | **437** | **540** | **581** | **613** | **Pailin** | **Kampong Seila** | **Chumkiri** | **Memut** | **Rattanakiri** |
| S | G | E | A/G | A | - | - | 2 | - | - |
| S | G | K/E | A/G | A | - | - | 2 | - | - |
| S | G | K/E | A | A | - | - | 1 | - | 1 |
| S | G | K/N | G | A | 1 | - | - | - | - |
| S | G | K/E/N | G | A | 1 | - | - | - | - |
| A | G | K/E | A | A | - | - | - | 2 | 2 |
| S/A | G | K/E | A | A | - | - | - | 1 | - |
| S | A/G | K | A | A | - | - | - | 3 | - |
| S/A | A/G | E | A | A | - | - | - | - | 1 |
| S/F | G | K | A | A | - | - | - | - | 1 |
| S/A | G | K | A | A | - | - | - | - | 1 |
| S/A | G | E/N | A/G | A | - | - | - | - | 1 |
| S/A | A/G | K | A | A | - | - | - | - | 1 |
| S/A | G | E | A | A | - | - | - | - | 1 |

*Note:* Mixed codons are shaded in gray.
