# Supplementary material for: Origin and Evolution of Sulfadoxine Resistant Plasmodium falciparum
Source: PLoS Pathog. 2010 Mar 26;6(3):e1000830. doi: 10.1371/journal.ppat.1000830 (PMC2847944; doi:10.1371/journal.ppat.1000830)
Supplement: Table S3 — The expected heterozygosity (H e) and number of alleles (A) at 10 microsatellite loci around dhps gene and at 8 loci on chromosomes 2 and 3 in Cambodia. (0.07 MB DOC) [file ppat.1000830.s003.doc]

**Table S3:** The expected heterozygosity (*H*_e_) and number of alleles (A) at 10 microsatellite loci around *dhps* gene and at 8 loci on chromosomes 2 and 3 in Cambodia.

| **Microsatellites** | **Wild type**  **(n = 20)** | | **Single mutant**  **(n = 17)** | | **Double mutant**  **(n = 37)** | | **Triple mutant**  **(n = 65)** | |
| --- | --- | --- | --- | --- | --- | --- | --- | --- |
|  | **A** | ***H_e_*±SD** | **A** | ***H_e_*±SD** | **A** | ***H_e_*±SD** | **A** | ***H_e_*±SD** |
| **Chr8/*dhps* loci** |  |  |  |  |  |  |  |  |
| -11 Kb | 3 | 0.19±0.10 | 3 | 0.23±0.11 | 2 | 0.48±0.03 | 3 | 0.50±0.03 |
| -7.5 Kb | 13 | 0.96±0.01 | 11 | 0.90±0.04 | 7 | 0.80±0.03 | 4 | 0.49±0.04 |
| -2.9 Kb | 5 | 0.73±0.05 | 3 | 0.58±0.05 | 4 | 0.68±0.02 | 5 | 0.51±0.04 |
| -1.5 Kb | 10 | 0.91±0.03 | 7 | 0.71±0.09 | 3 | 0.20±0.08 | 4 | 0.15±0.05 |
| -0.13 Kb | 2 | 0.48±0.06 | 2 | 0.51±0.03 | 2 | 0.15±0.07 | 1 | 0.00±0.00 |
| 0.03 Kb | 12 | 0.94±0.02 | 6 | 0.59±0.12 | 3 | 0.50±0.04 | 4 | 0.53±0.02 |
| 0.5 Kb | 7 | 0.84±0.03 | 4 | 0.63±0.06 | 3 | 0.50±0.04 | 2 | 0.06±0.04 |
| 1.4 Kb | 13 | 0.94±0.02 | 7 | 0.81±0.05 | 7 | 0.75±0.02 | 5 | 0.56±0.04 |
| 6.4 Kb | 9 | 0.92±0.02 | 5 | 0.70±0.08 | 5 | 0.75±0.02 | 4 | 0.50±0.03 |
| 9 Kb | 12 | 0.92±0.03 | 11 | 0.88±0.06 | 9 | 0.86±0.02 | 4 | 0.67±0.02 |
| **Mean** | **8.6±4.1** | **0.78±0.08** | **5.9±3.2** | **0.65±0.06** | **4.5±2.4** | **0.56±0.07** | **3.6±1.3** | **0.39±0.07** |
| **Chr2 loci*** |  |  |  |  |  |  |  |  |
| C2M27 | 10 | 0.91±0.03 | 11 | 0.95±0.01 | 13 | 0.90±0.01 | 13 | 0.83±0.03 |
| C2M29 | 6 | 0.70±0.07 | 6 | 0.65±0.10 | 10 | 0.71±0.06 | 9 | 0.57±0.06 |
| C2M34 | 13 | 0.95±0.01 | **7** | 0.85±0.03 | 14 | 0.90±0.02 | 15 | 0.88±0.01 |
| C2M33 | 9 | 0.90±0.02 | 11 | 0.94±0.02 | 16 | 0.93±0.01 | 15 | 0.89±0.01 |
| **Mean** | **9.5±2.8** | **0.86±0.05** | **8.7±2.6** | **0.85±0.06** | **13.2±2.5** | **0.86±0.05** | **13.0±2.8** | **0.79±0.07** |
| **Chr3 loci*** |  |  |  |  |  |  |  |  |
| C3M40 | 13 | 0.95±0.01 | 9 | 0.91±0.02 | 16 | 0.94±0.01 | 18 | 0.93±0.01 |
| C3M88 | 11 | 0.91±0.03 | 10 | 0.92±0.02 | 19 | 0.95±0.00 | 18 | 0.94±0.01 |
| C3M69 | 10 | 0.92±0.02 | 8 | 0.86±0.04 | 9 | 0.86±0.02 | 11 | 0.84±0.02 |
| C3M39 | 4 | 0.36±0.12 | 4 | 0.41±0.12 | 6 | 0.55±0.08 | 6 | 0.53±0.06 |
| **Mean** | **9.5±3.8** | **0.78±0.14** | **7.7±2.6** | **0.78±0.12** | **12.5±6** | **0.82±0.09** | **13.5±5.8** | **0.81±0.09** |

***Note:*** Wild type *dhps* codons [S_436_A_437_K_540_A_581_A_613_]. Single mutant [SGKAA, n = 16; and AAKAA, n = 1]. Double mutant [AGKAA, n = 10; FGKAA, n = 1; SGEAA, n = 14; and SGKGA, n = 12]. Triple mutant [AGEAA, n = 26; SGEGA, n = 11; SGNGA, n = 27; and AGKAT, n = 1]. The two isolates with quadruple *dhps* mutations were not analyzed here. A, Number of alleles per locus; *H_e_*, Expected heterozygosity; SD, Standard deviation.
