# Supplementary material for: Origin and Evolution of Sulfadoxine Resistant Plasmodium falciparum
Source: PLoS Pathog. 2010 Mar 26;6(3):e1000830. doi: 10.1371/journal.ppat.1000830 (PMC2847944; doi:10.1371/journal.ppat.1000830)
Supplement: Table S4 — Sample H e and sample FST at all 10 dhps and 8 neutral microsatellite loci as obtained in LOSITAN (0.04 MB DOC) [file ppat.1000830.s004.doc]

**Table S4:** Sample *H_e_* and sample F_ST_ at all 10 *dhps* and 8 neutral microsatellite loci as obtained in LOSITAN

| **Loci** | ***H_e_*** | **F_ST_** | ***P*^*^** |
| --- | --- | --- | --- |
| ***dhps* loci** |  |  |  |
| -11 Kb | 0.48 | 0.28 | **0.99989** |
| -7.5 Kb | 0.90 | 0.15 | **1** |
| -2.9 Kb | 0.76 | 0.19 | **0.999962** |
| -1.5 Kb | 0.73 | 0.34 | **1** |
| -0.13 Kb | 0.42 | 0.33 | **0.999965** |
| 0.03 Kb | 0.76 | 0.17 | **0.999894** |
| 0.5 Kb | 0.63 | 0.21 | **0.999998** |
| 1.4 Kb | 0.91 | 0.18 | **1** |
| 6.4 Kb | 0.85 | 0.17 | **1** |
| 9 Kb | 0.87 | 0.07 | **0.997353** |
| ***Neutral loci*** |  |  |  |
| C2M27 | 0.91 | 0.02 | 0.551725 |
| C2M29 | 0.67 | 0.03 | 0.666866 |
| C2M34 | 0.93 | 0.06 | **0.998506** |
| C2M33 | 0.93 | 0.03 | 0.854458 |
| C3M40 | 0.93 | 0.02 | 0.553196 |
| C3M88 | 0.94 | 0.03 | 0.804318 |
| C3M69 | 0.88 | 0.02 | 0.502992 |
| C3M39 | 0.47 | 0.03 | 0.679204 |

**P* (Simulated F_ST_<sample F_ST_). The outlier loci above the 0.95 quantile are shown in bold.
