# Supplementary material for: Origin and Evolution of Sulfadoxine Resistant Plasmodium falciparum
Source: PLoS Pathog. 2010 Mar 26;6(3):e1000830. doi: 10.1371/journal.ppat.1000830 (PMC2847944; doi:10.1371/journal.ppat.1000830)
Supplement: Table S5 — The 10-loci microsatellite haplotype profiles of the dhps alleles in Cambodia (0.04 MB PDF) [file ppat.1000830.s005.pdf]

Table S5: The 10-loci microsatellite haplotype profiles of the *dhps* alleles in Cambodia

	H	-11	-7.5	-2.9	-1.5	-0.13	Genotype	0.03	0.5	1.4	6.4	9	N
	H1	220	173	191	172	134	SAKAA	134	147	252	286	100	1
	H2	220	194	193	182	134	SAKAA	132	147	256	303	100	1
	H3	220	194	189	182	134	SAKAA	132	147	256	303	100	1
	H4	220	175	189	164	132	SAKAA	160	150	258	292	123	1
	H5	220	173	189	166	134	SAKAA	126	140	269	288	109	1
	H6	220	167	178	168	134	SAKAA	147	143	266	288	120	1
	H7	220	183	189	170	132	SAKAA	156	147	252	295	118	1
	H8	220	163	191	174	134	SAKAA	138	147	254	297	115	1
	H9	220	173	193	178	132	SAKAA	132	148	280	299	100	1
A	H10	224	181	189	170	134	SAKAA	126	145	254	294	114	1
	H11	220	172	189	168	134	SAKAA	145	147	274	286	114	1
	H12	220	169	193	188	132	SAKAA	141	145	261	295	115	1
	H13	228	181	191	168	134	SAKAA	136	143	252	310	119	1
	H14	220	161	189	166	132	SAKAA	126	145	254	299	109	1
	H15	220	153	191	166	134	SAKAA	128	146	257	299	100	1
	H16	220	165	191	168	134	SAKAA	130	145	273	297	122	1
	H17	220	169	193	172	134	SAKAA	136	146	254	286	106	1
	H18	220	183	189	172	134	SAKAA	128	150	255	286	113	1
	H19	220	177	189	165	132	SAKAA	128	150	257	303	115	1
	H20	220	161	183	168	132	SAKAA	130	145	263	292	110	1
	H21	220	163	183	168	132	SGKAA	126	145	277	292	111	2
	H22	220	163	183	168	132	SGKAA	126	145	277	292	109	1
	H23	220	163	183	168	132	SGKAA	128	145	277	292	109	1
	H24	220	163	183	168	132	SGKAA	126	145	277	286	102	1
	H25	220	195	183	168	132	SGKAA	126	145	277	292	109	1
	H26	220	173	193	168	132	SGKAA	126	145	268	292	109	1
	H27	220	165	189	168	134	SGKAA	126	150	248	295	91	1
B	H28	216	153	189	178	134	SGKAA	126	150	248	295	92	1
	H29	220	182	189	172	134	SGKAA	126	150	248	299	93	1
	H30	220	165	189	172	134	SGKAA	126	150	248	295	109	1
	H31	220	175	189	172	134	SGKAA	132	150	248	292	109	1
	H32	224	179	189	166	132	SGKAA	143	145	275	292	110	1
	H33	220	169	189	196	132	SGKAA	138	139	266	297	100	1
	H34	220	171	183	176	132	SGKAA	130	146	263	297	97	1
	H35	220	167	183	170	134	SGKAA	126	150	256	286	113	1
	H47	216	153	191	172	134	SGKGA	141	145	252	286	100	4
	H48	216	153	191	172	134	SGKGA	141	145	252	286	102	6
	H49	216	153	189	172	134	SGKGA	141	145	252	286	100	1
	H50	216	152	191	172	134	SGKGA	141	146	251	285	100	1
	H47	216	153	191	172	134	SGEGA	141	145	252	286	100	5
	H48	216	153	191	172	134	SGEGA	141	145	252	286	102	3
	H72	220	153	191	172	134	SGEGA	141	145	252	286	100	2
	H73	220	167	191	172	134	SGEGA	141	145	252	285	102	1
	H47	216	153	191	172	134	SGNGA	141	145	252	286	100	4
C	H48	216	153	191	172	134	SGNGA	141	145	252	286	102	4
	H74	216	153	191	172	134	SGNGA	141	145	252	299	100	4
	H75	216	153	191	172	134	SGNGA	141	145	252	299	102	5
	H76	216	153	191	172	134	SGNGA	141	145	252	299	109	3
	H77	216	153	191	174	134	SGNGA	141	145	252	286	102	2
	H78	220	167	191	172	134	SGNGA	141	145	252	286	100	1
	H79	216	153	191	172	134	SGNGA	156	145	227	301	102	1
	H80	216	153	191	172	134	SGNGA	126	145	252	299	102	1
	H81	216	153	195	172	134	SGNGA	141	145	252	286	102	1
	H82	216	153	180	172	134	SGNGA	141	145	250	299	100	1
	H37	220	167	183	172	134	AGKAA	126	145	250	299	109	2
	H38	216	173	189	172	134	AGKAA	126	145	250	299	109	1
	H39	220	167	193	172	134	AGKAA	126	145	250	299	109	1
	H40	220	175	183	172	134	AGKAA	126	145	250	299	109	1
	H41	220	167	183	170	132	AGKAA	126	145	250	299	109	1
	H42	220	167	183	172	134	AGKAA	126	145	250	292	126	1
	H43	220	167	183	172	134	AGKAA	126	145	250	292	110	1
	H44	220	175	183	169	132	AGKAA	126	150	279	292	111	1
	H45	220	153	191	170	134	AGKAA	126	145	227	292	123	1
	H59	216	153	183	172	134	AGEAA	126	145	250	299	109	3
	H60	216	153	183	172	134	AGEAA	126	145	251	299	109	1
D	H61	216	167	183	172	134	AGEAA	126	145	250	299	109	1
	H62	218	153	183	172	134	AGEAA	126	145	250	299	109	1
	H37	220	167	183	172	134	AGEAA	126	145	250	299	109	9
	H63	220	167	191	172	134	AGEAA	126	145	250	299	109	3
	H64	220	178	189	172	134	AGEAA	126	145	252	299	109	1
	H65	220	167	183	172	134	AGEAA	126	143	250	299	109	1
	H66	220	167	183	176	134	AGEAA	126	143	250	299	109	1
	H67	220	167	183	174	134	AGEAA	126	145	250	299	109	1
	H68	220	177	183	166	134	AGEAA	126	145	250	299	109	1
	H69	220	167	191	172	134	AGEAA	126	145	250	299	110	1
	H70	220	167	183	172	134	AGEAA	126	145	227	299	109	1
	H71	220	153	183	172	134	AGEAA	127	145	227	286	109	1
	H51	220	167	189	172	134	SGEAA	126	145	250	299	109	2
	H52	220	173	189	172	134	SGEAA	126	150	248	295	93	5
	H53	220	176	189	172	134	SGEAA	126	150	248	295	92	2
	H54	216	153	191	172	134	SGEAA	141	145	272	286	100	1
E	H55	220	167	189	172	134	SGEAA	126	150	248	295	93	1
	H56	220	194	189	172	134	SGEAA	126	150	248	295	93	1
	H57	220	194	189	172	134	SGEAA	132	150	248	295	92	1
	H58	220	194	189	172	134	SGEAA	126	150	248	286	102	1
	H84	216	153	183	172	134	AGEAT	126	150	272	292	111	1
	H85	216	153	191	172	134	FGEAT	126	150	272	292	111	1
F	H36	220	165	183	168	134	AAKAA	132	145	266	292	119	1
	H83	220	167	183	172	134	AGKAT	126	145	248	299	109	1
	H46	220	175	183	169	132	FGKAA	126	150	252	295	110	1

Note: Identical colors (shaded boxes) in the haplotype column represent proposed common lineages. The wild type (SAKAA) in panel A and single mutant (SGKAA) in panel B have multiple unique haplotypes backgrounds (as indicated by scattered pink, gray and blue shading) and contain allele sizes present in the C, D, E and F panels. The single mutant SGKAA lineage gives rise to the double mutant SGKGA lineage (predominantly pink shaded haplotypes in panel C) which in turn gives rise to two triple mutants, SGEGA and SGNGA. Lineage B also gives rise to the double mutant AGKAA which is a precursor for the triple mutant AGEAA (predominantly gray shaded haplotypes in panel D). Lineage B also gives rise to the third double mutant, SGEAA which is shaded predominantly blue (Panel E). Panel F has limited and rare *dhps* alleles with different haplotype backgrounds. H, Haplotype; N, number of isolates sharing a particular haplotype. The list of haplotypes in panels C, D, E and F are not in any particular order.
