# Supplementary figures and images for: Origin and Evolution of Sulfadoxine Resistant Plasmodium falciparum
Source: PLoS Pathog. 2010 Mar 26;6(3):e1000830. doi: 10.1371/journal.ppat.1000830 (PMC2847944; doi:10.1371/journal.ppat.1000830)

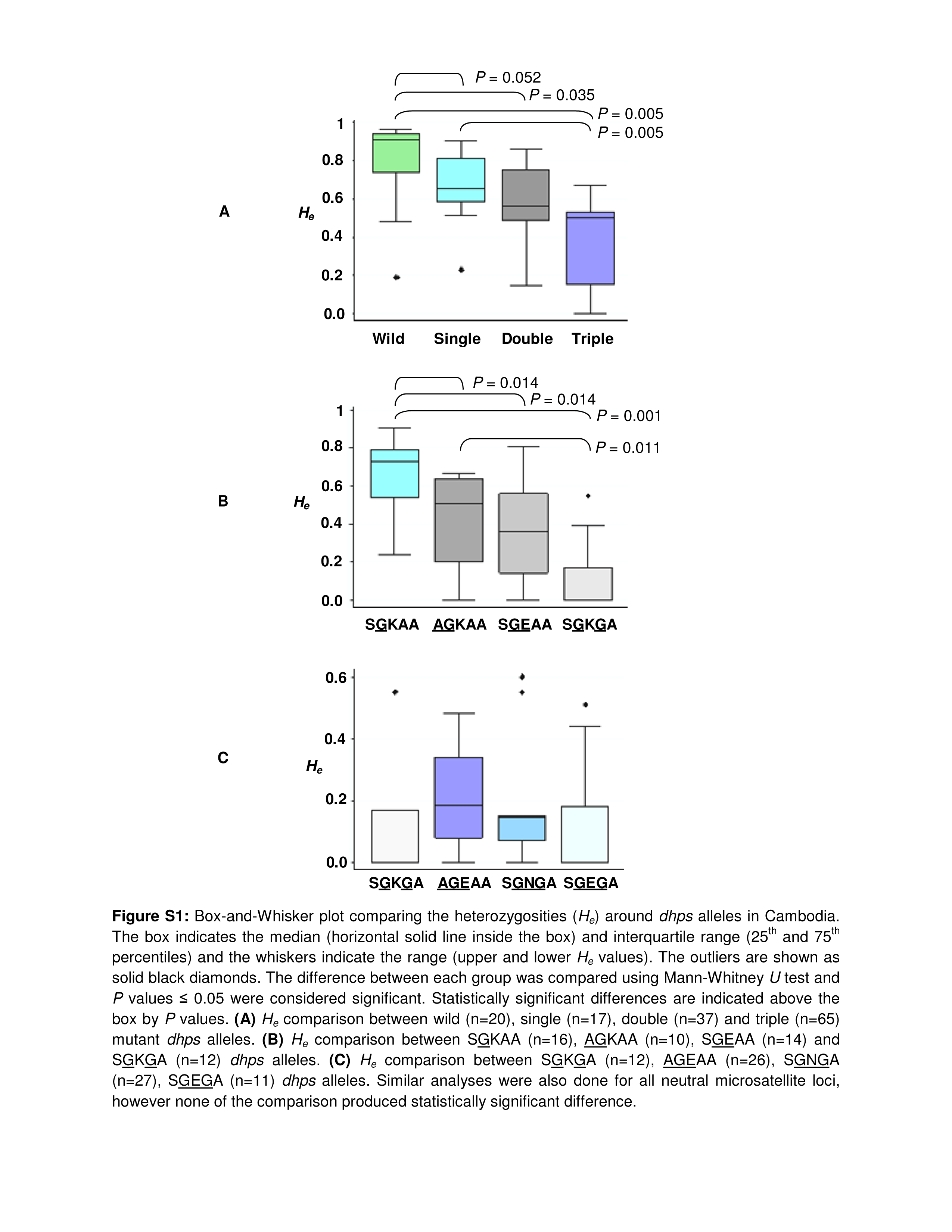

Supplement: Figure S1 — Box-and-Whisker plot comparing the heterozygosities (H e) around dhps alleles in Cambodia. The box indicates the median (horizontal solid line inside the box) and interquartile range (25th and 75th percentiles) and the whiskers indicate the range (upper and lower H e values). The outliers are shown as solid black diamonds. The difference between each group was compared using Mann-Whitney U test and P values≤0.05 were considered significant. Statistically significant differences are indicated above the box by P values. (A) H e comparison between wild (n = 20), single (n = 17), double (n = 37) and triple (n = 65) mutant dhps alleles. (B) H e comparison between SGKAA (n = 16), AGKAA (n = 10), SGEAA (n = 14) and SGKGA (n = 12) dhps alleles. (C) H e comparison between SGKGA (n = 12), AGEAA (n = 26), SGNGA (n = 27), SGEGA (n = 11) dhps alleles. Similar analyses were also done for all neutral microsatellite loci, however none of the comparison produced statistically significant difference. (0.94 MB TIF) [file ppat.1000830.s006.tif]
